# Decitabine inhibits T cell proliferation via a novel TET2-dependent mechanism and exerts potent protective effect in mouse auto- and allo-immunity models

**DOI:** 10.18632/oncotarget.18063

**Published:** 2017-05-22

**Authors:** Xue Wang, Jun Wang, Yong Yu, Tonghui Ma, Ping Chen, Bing Zhou, Ran Tao

**Affiliations:** ^1^ Shanghai Institute of Immunology, Shanghai Jiao Tong University School of Medicine, Shanghai, PR China; ^2^ Department of Thoracic Surgery, Hangzhou Municipal Hospital of Traditional Chinese Medicine, Hangzhou, PR China; ^3^ Max Delbrück Center for Molecular Medicine, Berlin, Germany; ^4^ Provincial Key Laboratory of Cardiac Transplantation, Zhejiang Provincial People's Hospital (ZJPPH), Hangzhou, PR China; ^5^ Department of Obstetrics & Gynecology, Shaoxing Second Municipal Hospital, Shaoxing, PR China; ^6^ Department of Cardiothoracic Surgery, Zhejiang Provincial People's Hospital (ZJPPH), Hangzhou, PR China; ^7^ Department of Hepatobiliary-Pancreatic & Minimally Invasive Surgery, Zhejiang Provincial People's Hospital (ZJPPH), Hangzhou, PR China

**Keywords:** decitabine, experimental autoimmune encephalomyelitis, cardiac transplantation, T cell proliferation, TET2

## Abstract

Multiple sclerosis (MS) is an autoimmune disease characterized by the dysregulated immune response including innate and adaptive immune responses. Increasing evidence has proven the importance of epigenetic modification in the progression of MS. Recent studies revealed that low-dose decitabine (Dec, 5-Aza-2′-deoxycytidine), which incorporates into replicating DNA and inhibits DNA methylation, could prevent experimental autoimmune encephalomyelitis (EAE) development by increasing the number of regulatory T cells (Tregs). Here, we showed that higher-dose decitabine relative to previous studies could also distinctly protect mice from EAE and allogeneic cardiac transplantation. Mechanistic studies revealed decitabine suppressed innate responses in EAE mice through inhibiting the activation of microglia and monocyte-derived macrophages that contributed to reduce the severity of EAE. Furthermore, differentiation of naïve CD4^+^ T cells into Th1 and Th17 cells was significantly suppressed by decitabine *in vivo* and *in vitro*. Though *in vitro* studies showed decitabine could induce Treg differentiation, there was no obvious change in the percentage of Tregs in Dec-treated EAE mice. Most importantly, we found that T cell proliferation was potently inhibited *in vivo* and *in vitro* by higher-dose decitabine through increased gene expression of the DNA dioxygenase TET2 which facilitated the expression of several cell cycle inhibitors. Collectively, our study provides novel mechanistic insights of using the epigenetic modifying agents in the management of both allo- and auto-immune responses.

## INTRODUCTION

Multiple sclerosis (MS) is a chronic inflammatory, demyelinating disease of the central nervous system (CNS), affecting approximately 2.5 million individuals worldwide. It manifests as neurological deficits that frequently exhibit a relapsing-remitting pattern, and can either resolve completely or leave residual deficits. A complicated inflammatory cascade involving both the innate and adaptive immune responses is believed to initiate and control the disease progression [1, 2]. So far there is no effective therapeutic means in the management of MS. Currently available disease-modifying drugs like interferon-β (IFN-β), glatiramer acetate, fingolimod, etc, have either limited efficacy or serious side effects [1, 3]. Thus, there is still an urgent need to develop more effective and safer drugs for the treatment of MS.

Due to the similarity of key pathological features of MS and the well-established mouse model of experimental autoimmune encephalomyelitis (EAE), such as demyelination, inflammation, axonal loss and gliosis, EAE has become the most commonly used experimental model for investigating the mechanisms of MS, as well as for therapeutic development [4]. It is widely believed that autoreactive T cells, myeloid cells and resident microglial cells are obligatory requirements for induction of CNS pathogenesis of MS. As the major components of the innate immune system, microglia and monocyte-derived macrophages constitute the first-line barrier of CNS. Microglia, which originate from the yolk sac and migrate into the CNS during early embryogenesis and acquire properties like production of cytokines and chemokines, phagocytosis, and antigen presentation [5, 6], are exquisitely alert to brain injury and disease [7]. Microglia are regarded as liaisons between the immune system and CNS, and participate in the regulation of inflammation in MS [8]. The process of microglia activation starts early in the pathological process of EAE, before the entry of peripheral monocyte/macrophage into the CNS and appearance of clinical symptoms [8, 9]. Monocyte-derived macrophages which are indistinguishable from microglia at the level of morphology and surface markers, have been proved to be associated with EAE exacerbation. Elimination of macrophages by means of silica dust or liposomes containing dichloromethylene diphosphonate remarkably attenuates the clinical signs of EAE [10–12]. Activated microglia and macrophages, which are hallmarks of neuroinflammation, may contribute to CNS damage and chronic neurodegeneration through the release of harmful cytokines and chemokines, enhancement of antigen presentation via upregulation of MHC class II molecules, etc [13]. As the core of the adaptive immunity, naive CD4^+^ T cells display a high degree of plasticity and the ability to differentiate into effector (such as Th1 and Th17) and suppressor (such as Treg) sublineages in response to various developmental and environmental stimuli [14]. Th1 and Th17 cells have been widely recognized as central players in the pathogenesis of MS, while Tregs are essential in the fine-tune of immune activation and confer protection against MS.

Decitabine (5-Aza-2′-deoxycytidine), a FDA-approved hypomethylating agent, is a chemical analogue of cytidine and can incorporate into replicating DNA, where it irreversibly inactivates DNMT1 and inhibits DNA methylation. At present, decitabine has been successfully used to treat myelodysplastic syndrome (MDS) through inhibiting cell proliferation by blocking DNA synthesis and promoting cell differentiation via inducing hypomethylation [15]. More recently, increasing evidence showed that decitabine could induce genetically and functionally Tregs through Foxp3 demethylation, and prevent the occurrence of autoimmune diseases [16–19]. Two recent studies suggested that decitabine could increase the percentage and number of Tregs in EAE mice at low dosage (0.15 mg/kg/d), which was thought to be essential to protect mice from EAE [20, 21]. However, in the treatment of MDS decitabine displays a dual mechanism of action depending on dose. Lower dose is associated with demethylating activity through promoting cell differentiation and expression of tumor suppressor genes, while higher dose is cytotoxic by inhibiting cell proliferation through trapping DNA methyltransferase and blocking DNA synthesis [22]. To further explore the immnuo-regulatory effect of decitabine, we attempted to increase the dosage of decitabine in EAE and cardiac transplantation models, and the mechanism involved was also studied.

## RESULTS

### Decitabine protects mice from MOG-induced EAE and inhibits the proinflammatory response of CNS

**Figure 1:**
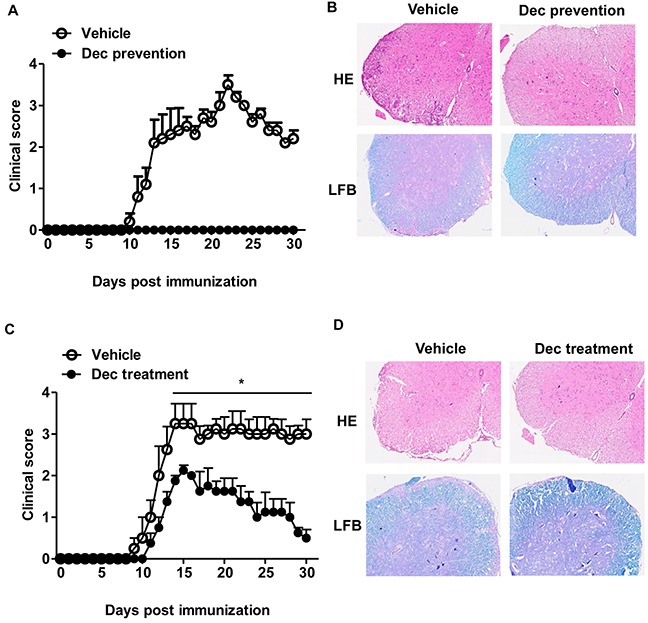
Systemic administration of decitabine is exquisitely protective against EAE Clinical scores of EAE mice received either vehicle or decitabine (0.25 mg/kg/day, intraperitoneal injection) treatment for 14 days with the preventive **(A)** or treatment **(C)** protocols (n=5 mice per group). Data were expressed as mean±S.E.M. Spinal cord sections were stained with H&E or LFB at day 18 for prevention protocol **(B)** or day 30 for treatment protocol **(D)** after immunization (original magnification, ×40). *P < 0.05.

To explore the potential therapeutic effects of decitabine on MOG-induced-EAE, peptide MOG35-55-immunized C57BL/6 mice were treated with decitabine (0.25 mg/kg) for 2 consecutive weeks (from day 3 to day 16 after immunization as preventive protocol or from day 10 to day 23 as a treatment protocol). Decitabine showed potently preventive as well as therapeutic effects on EAE, in particular, it completely prevented the onset of EAE (Figure 1A, 1C). All mice well tolerated the relatively higher dose of decitabine treatment, because there was no change in behavior or fur appearance, and body weight gain was observed in EAE mice treated with decitabine using the preventive protocol (Supplementary Figure 1). Consistent with the clinical results, decitabine treated EAE mice exhibited much less inflammatory cell infiltration and fewer demyelination of spinal cord compared with vehicle-treated mice upon pathological examination (Figure 1B, 1D). The level of peripheral blood cells could provide information about the presence of inflammation induced by infection, autoimmune diseases or allergy. Our study suggested that white blood cells, particularly lymphocyte subclasses, were lower in decitabine treated EAE mice with the treatment protocol as compared with the vehicle group (Supplementary Table 1). These results supported that decitabine was readily effective in the prevention and treatment of EAE in mice.

**Figure 2:**
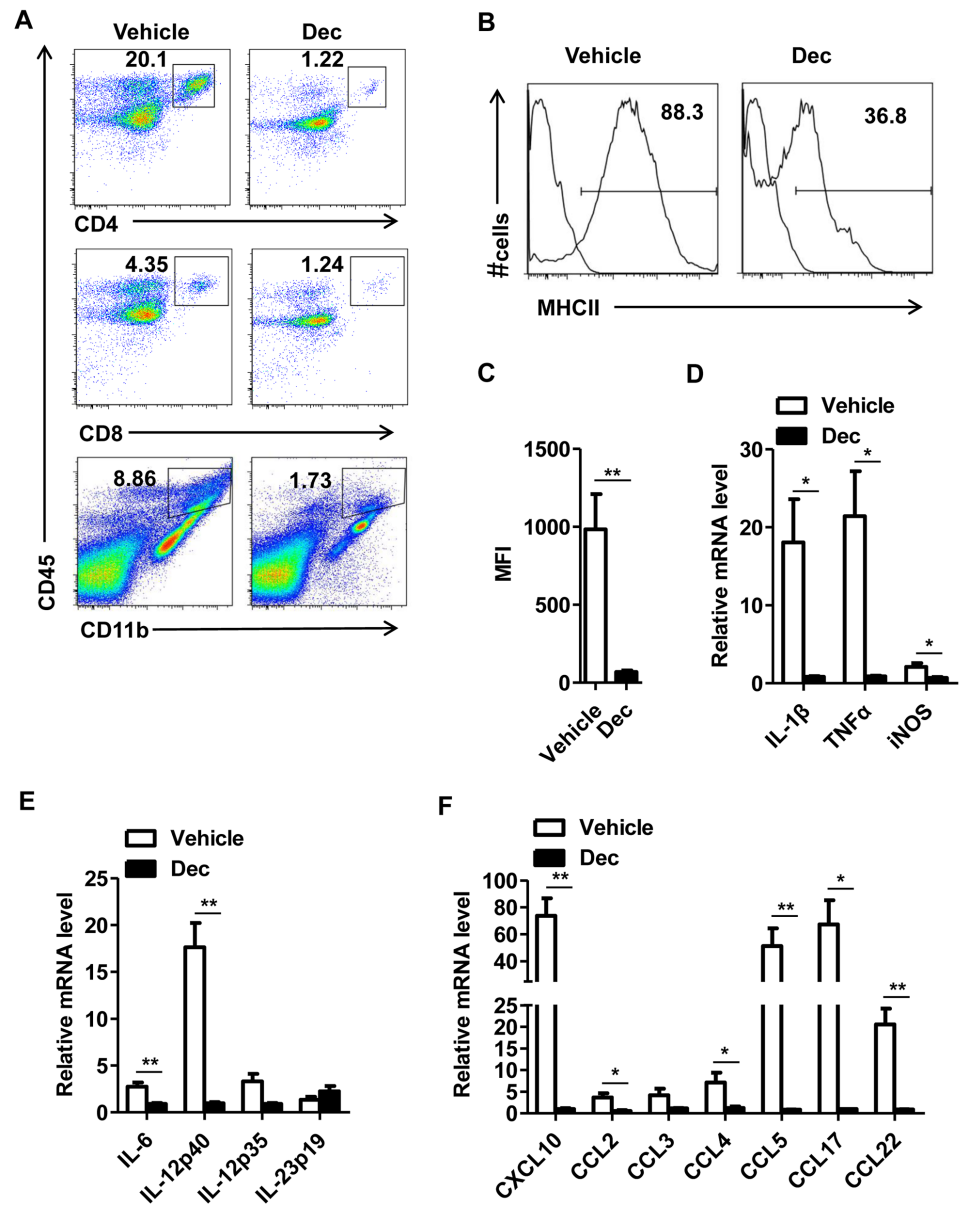
Decitabine inhibits proinflammatory response of CNS in EAE mice CNS (brain and spinal cord) mononuclear cells from EAE mice treated with vehicle or decitabine at day18 after immunization were assessed by flow cytometry **(A).** The expression of MHC-II on the CD11b^+^ population was analyzed. Results were presented as representative plot **(B)**, and histogram with MFI level **(C).** Quantification of mRNA expression of proinflammatory cytokines and chemokines in spinal cords of EAE mice treated with vehicle or decitabine (n=5 mice per group) at day 18 after immunization **(D, E, F).** Data **(C, D, E, F)** were expressed as mean±S.E.M. *P < 0.05, **P < 0.01.

*Ex-vivo* data showed that decitabine-treated EAE mice were found to have lower frequencies of CNS-infiltrating inflammatory T cells (CD4^+^CD45^hi^ and CD8^+^CD45^hi^), macrophages and activated resident microglial cells (CD11b^+^CD45^hi^) (Figure 2A). During the course of EAE, activated microglia upregulated surface expression of CD45 and MHC class II [5]. Along this line, decitabine-treated EAE mice showed lower expression of MHC class II presented by percentage and mean fluorescence intensity (MFI) as compared with the vehicle group (Figure 2B, 2C), indicating that decitabine could inhibit microglia activation. It is known that proinflammatory cytokines and chemokines play crucial roles in inflammation and immune cell recruitment. Notably, we found that decitabine significantly reduced the expression of IL-1, TNF-α, iNOS, which readily caused CNS damage per se, as well as a panel of inflammatory cytokines and chemokines like CXCL10, CCL2, CCL3, CCL4, CCL5, CCL17, CCL22, IL-6, IL-12 and IL-23, which were associated with T cell recruitment and differentiation (Figure 2D, 2E, 2F). These data collectively indicated that decitabine downregulated the expression of key mediators related to CNS inflammation, leading to decreased inflammatory infiltration of CNS in the pathogenesis of EAE.

### Decitabine inhibits allograft rejection and T cell allo-immunity in mouse cardiac transplantation

**Figure 3:**
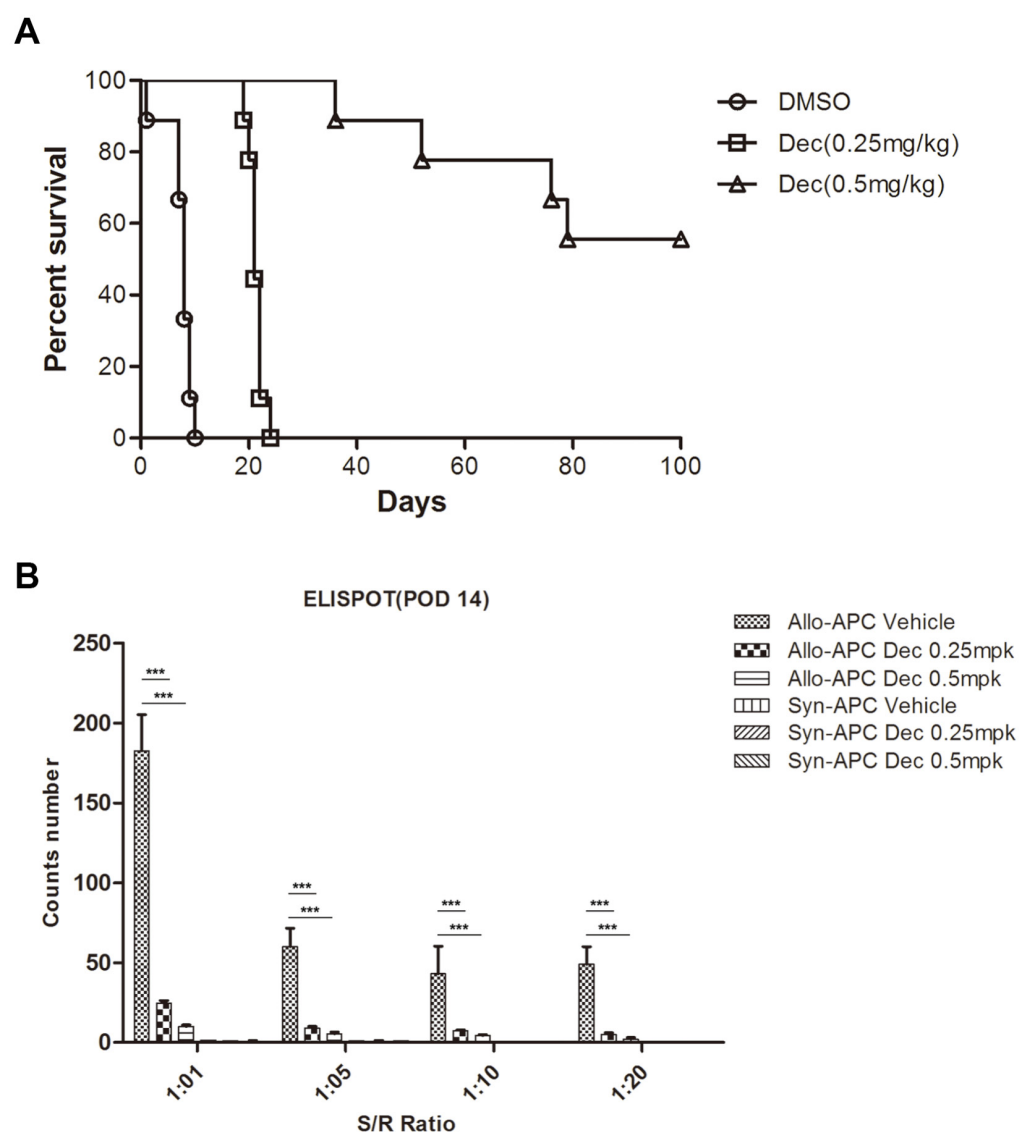
Decitabine inhibits cardiac allograft rejection and donor-specific T cell response **(A)** Kaplan-Meier survival analysis of cardiac allografts with or without decitabine treatment (0.25 or 0.5 mg/kg/day, intraperitoneal injection). **(B)** Allogeneic T cell immunity in cardiac transplantation mice treated with vehicle or decitabine was evaluated by monitoring the alloantigen-specific IFN-γ production via ELISPOT assay. Data **(A, B)** were expressed as mean±S.E.M. ***P < 0.001.

In order to test whether decitabine plays a similarly protective role in other immune-related models, we performed mouse cardiac transplantation across the MHC barrier (BALB/c→B6). Those recipient mice were treated with either decitabine (0.25 or 0.5 mg/kg/d) or vehicle for 14 consecutive days starting from post-operative day 1. We found that decitabine (0.25 mg/kg/d) significantly prolonged allograft survival in comparison to that of the vehicle-treated group (MST= 21.8 vs. 8.7 days, p=0.000). More importantly, further increase of decitabine dose (0.5 mg/kg/d) could induce permanent allograft survival (MST= 100 vs. 8.7 days, p=0.000) (Figure 3A). To further evaluate the potency of the recipient-anti-donor immune response, we used enzyme-linked immunosorbent spot (ELISPOT) assays to quantify donor-specific IFN-γ –producing cells in the recipient spleen. We found that decitabine-treated recipients using either regimen (0.25 or 0.50 mg/kg/d) had obviously fewer donor-responsive T cells than vehicle-treated control animals (Figure 3B), suggesting that decitabine exerted potent immunosuppressive effect in allogeneic immune response.

### Decitabine regulates T cell differentiation

**Figure 4:**
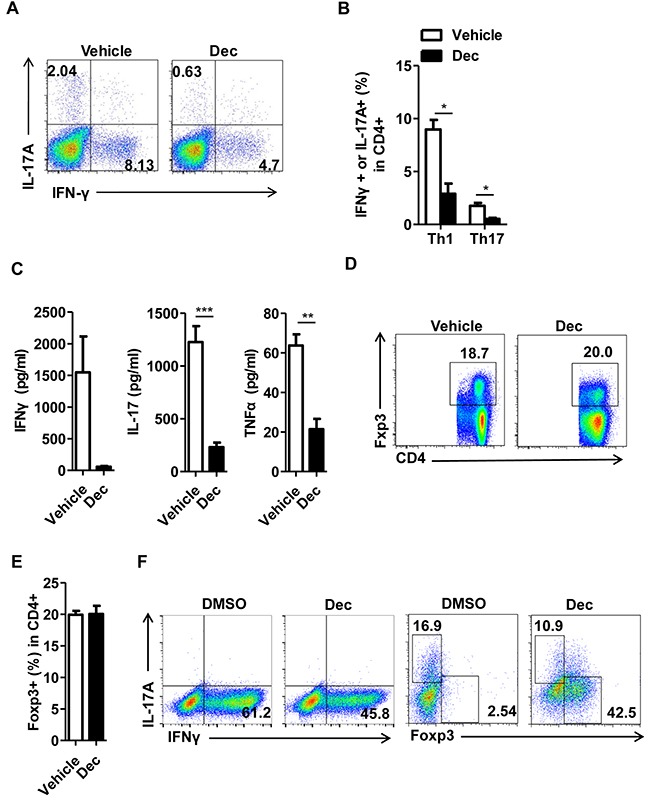
Decitabine modulates T cell differentiation *in vivo* and *in vitro* Splenocytes were harvested from EAE mice treated with vehicle or decitabine at day18 after immunization. The frequency of Th1 and Th17 in splenocytes was analyzed by FACS **(A, B).** Splenocytes were restimulated with MOG35–55 peptide (25 μg/ml), and culture supernatants were collected at 72 h for cytokine measurement by ELISA **(C).** The frequency of Treg population in splenocytes was analyzed by FACS **(D, E).** The effect of decitabine on Th1, Th17, Treg differentiation *in vitro* was analyzed by FACS **(F).** Data **(B, C, E)** were expressed as mean±S.E.M. *P < 0.05, **P < 0.01, ***P < 0.001.

Since T cell subsets are well-known players that orchestrate the pathogenesis of CNS inflammation in EAE mice and MS patients, we next examined whether decitabine could modulate T cell differentiation. We first isolated splenocytes from vehicle- or decitabine-treated EAE mice, and analyzed the changes of CD4^+^ T cell subsets. The percentages and absolute numbers of Th1 and Th17 cells were markedly decreased upon decitabine treatment (Figure 4A, 4B, Supplementary Figure 2), consistent with lower production of proinflammatory cytokines characteristic of EAE, including IFN-γ, IL-17, and TNF-α (Figure 4C). Unexpectedly, the percentage of Foxp3^+^ Tregs was not significantly affected, and even the absolute number was decreased by our decitabine treatment protocol (Figure 4D, 4E, Supplementary Figure 2). To further confirm the effect of decitabine on the differentiation of T cells, we adopted *in vitro* cell differentiation models and found that differentiation of naïve CD4^+^ T cells into Th1 and Th17 cells was significantly suppressed in the presence of 1 μM decitabine under Th1 and Th17 polarizing conditions, respectively (Figure 4F), which was in agreement with the *in vivo* results. However, the expression of Foxp3 was significantly increased in the presence of 1 μM decitabine compared with control (Figure 4F), though *in vivo* studies showed no obvious change.

### Decitabine inhibits T cell proliferation *in vitro* and *ex-vivo*

**Figure 5:**
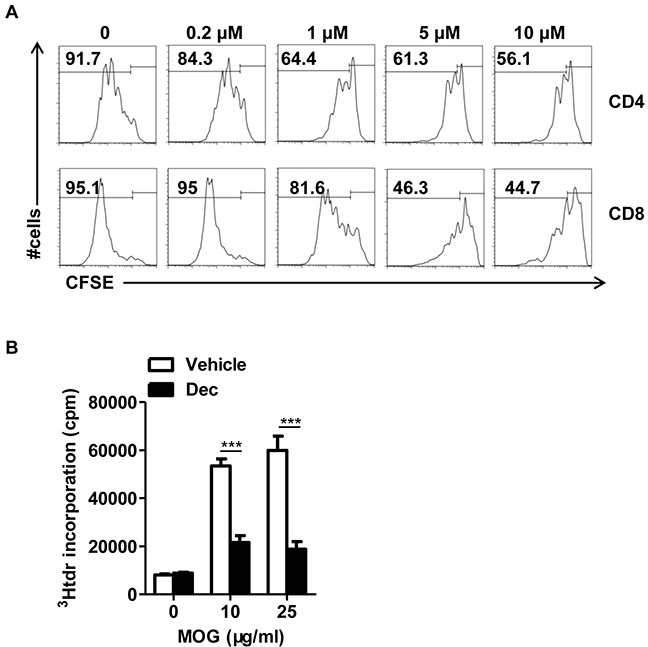
Decitabine inhibits naïve T proliferation *in vitro* and modulated MOG-reactive CD4^+^T cell response *in vivo* The effect of decitabine on the proliferation of T cell subsets stimulated with anti-CD3 plus anti-CD28 mAbs at different concentrations was examined by CFSE-based assay **(A).** Splenocytes from EAE mice treated with or without decitabine were restimulated ex vivo with indicated concentrations of the MOG35–55 peptide, and cell proliferation was assessed by [^3^H] thymidine incorporation assay **(B).** All data were expressed as mean±S.E.M. ***P < 0.001.

Despite the multifaceted roles of decitabine on innate and adaptive immune responses, surprisingly few studies have shed light on its influence on effector T cell activation, which is the cornerstone of the pathogenesis in both auto- and allo-immunities. Therefore, we attempted to explore the effect of decitabine on T cell proliferation and the underlying mechanism. Splenocytes from wild-type mice were stimulated with anti-CD3/anti-CD28 monoclonal antibodies (mAbs) in the presence of decitabine at various concentrations. We observed a strong dose-dependent inhibition of the proliferation in both CD4^+^ and CD8^+^ subsets, as judged by the CFSE profile (Figure 5A). This inhibitory effect was not attributed to induction of cell apoptosis (Supplementary Figure 3). To further determine whether decitabine could inhibit antigen-specific T cell response, we investigated the response of encephalitogenic T cells to MOG peptide ex vivo using [3H] thymidine incorporation assay. The proliferation of encephalitogenic T cells in decitabine-treated mice was much weaker than the vehicle-treated group upon MOG peptide re-stimulation *in vitro* (Figure 5B).

### The inhibition of T cell proliferation by decitabine is associated with increased expression of several cell cycle inhibitors

**Figure 6:**
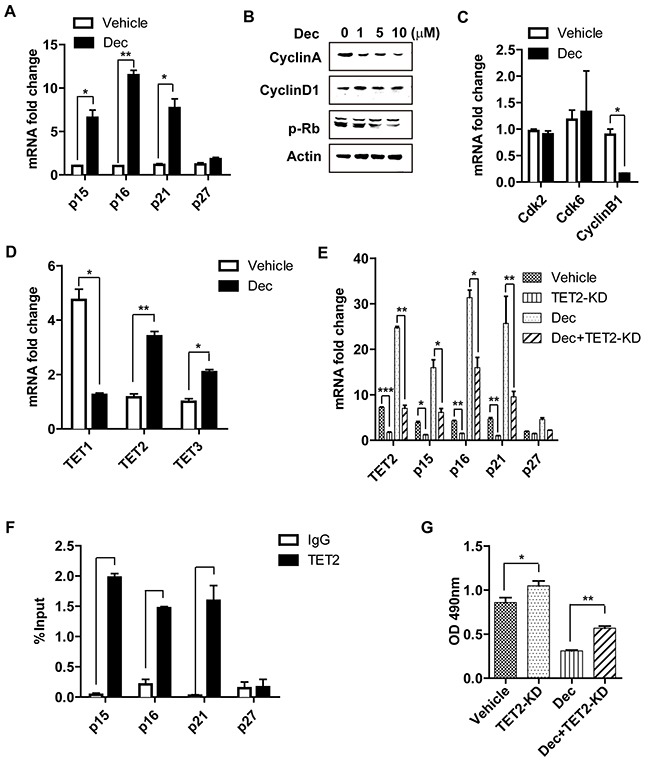
The inhibition of decitabine on T cell proliferation is associated with increased expression of several cell cycle inhibitors and TET2 Decitabine (10 μM) promoted the mRNA expression of several cell cycle inhibitors (p15, p16, p21, p27) upon T cell activation **(A).** The effect of decitabine (10 μM) on the cell cycle progression of naïve CD4^+^ T cell stimulated with anti-CD3 plus anti-CD28 mAbs at different concentrations was analyzed by western blot **(B)** and real-time PCR **(C).** Decitabine (10 μM) promoted the expression of TET2 and TET3 but reduced TET1 expression in naïve CD4^+^ T cells stimulated with anti-CD3 plus anti-CD28 mAbs *in vitro* **(D).** After knocking down the expression of TET2 in naïve CD4^+^ T cells, these cells were treated with vehicle or decitabine (10 μM). The different impacts on the transcriptional level of cell cycle inhibitors (p15, p16, p21, p27) **(E)** and the rate of cell proliferation **(G)** were shown. **(F)** ChIP assays of naïve CD4^+^ T cells, using an anti-TET2 antibody and PCR primers specific for promoters of *p15*, *p16*, *p21* and *p27*. IgG antibody was used as negative control and input DNA was used as internal control. Data **(A, C, D, E, F, G)** were expressed as mean±S.E.M. *P < 0.05, **P < 0.01, ***P < 0.001.

To further explore the mechanism through which decitabine inhibited the proliferation of T cells, the expression of a panel of cell cycle–related proteins which are known to be involved in the regulation of lymphocyte proliferation [24, 25] were examined. Compared with vehicle-treated CD4^+^ naïve counterparts, quantitative PCR revealed significantly increased expression of several key cell cycle inhibitors (p15, p16, p21 but not p27) in those naïve T cells underwent decitabine treatment (Figure 6A).

In addition to the cyclin dependent kinase inhibitors, other cell cycle-related proteins (such as cyclin-dependent kinases and cyclins) also control cell proliferation via regulating cycle progression [26]. Compared with vehicle controls, there was significantly reduced expression of Cyclin A, phosphorylated retinoblastoma protein(p-Rb) and Cyclin B1 (Figure 6B, 6C) in decitabine-treated T cells, while no difference in the expression of Cyclin D1, cyclin-dependent kinase 2 (Cdk2) and cyclin-dependent kinase 6 (Cdk6) after decitabine treatment was observed (Figure 6B, 6C).

### The inhibition of T cell proliferation by decitabine is associated with increased expression of TET2

The ten-eleven-translocation (TET) family proteins including TET1, TET2 and TET3 belong to the α-ketoglutarate and Fe^2+^-dependent enzymes, which can alter the methylation status of DNA by catalyzing the conversion of 5-methyl-cytosine (5-mC) to 5-hydroxymethyl-cytosine (5-hmC) and promote DNA demethylation [27]. TET2 is the most abundant TET protein in hematopoietic cells [28]. Decitabine is a well-recognized DNMT inhibitor, however, whether it also has an impact on TET family proteins remains obscure. RT-qPCR showed that in CD4^+^ naïve T cells stimulated with anti-CD3 and anti-CD28 antibodies, decitabine could significantly upregulate the expression of TET2 and TET3, but reduce that of TET1 (Figure 6D). To study the functions of TET proteins, we knocked-down TET1, TET2, and TET3. Interestingly, knockdown of TET2 reduced the expression of p15, p16 and p21 in T cells, but had little impact on that of p27 (Figure 6E). More importantly, while decitabine treatment greatly boosted the expression of p15, p16 and p21, such effect was significantly diminished when TET2 was knocked down, suggesting this effect is partially dependent on TET2. In contrast, no obvious changes of these cell cycle inhibitors could be observed when TET1 or TET3 was knocked-down in either vehicle-treated or decitabine-treated CD4^+^ naïve T cell (Supplementary Figure 4A, 4B). To confirm the direct effects of TET2 on the gene transcription of p15, p16, p21 and p27, we performed ChIP assay using TET2 antibody. Our results showed that in naïve T cells, TET2 was enriched in promoters of p15, p16 and p21 genes, but not in p27 (Figure 6F). We further investigated the effect of TET proteins on the proliferation of CD4^+^ naïve T cells. Consistent with the aforementioned results, knocking-down TET2 clearly promoted the proliferation of CD4^+^ naïve T cells either in decitabine or vehicle-treated group (Figure 6G), while knockdown of TET1 or TET3 had minimal impact on CD4^+^ T cell proliferation (Supplementary Figure 4C, 4D). Taken together, our data revealed that decitabine treatment increased the expression of TET2, which regulated the transcription of certain cell cycle inhibitors (p15, p16 and p21) and inhibition of CD4^+^ naïve T cell proliferation *in vitro*.

## DISCUSSION

In this work, we proved that the methylation-modifying drug decitabine could become a promising therapeutic strategy in the treatment of MS as well as in the prevention of cardiac allograft rejection. However, other than previous studies which claimed the protective effect by decitabine in EAE was mainly attributed to the induction of Tregs, we provided solid evidence that multifaceted mechanisms were involved in this overwhelmingly immunosuppressive effect by decitabine. Decitabine was capable of inhibiting both innate and adaptive immunity to hinder the progression of EAE. Furthermore, increasing the dosage of decitabine would not further increase the proportion and/or absolute number of Tregs as expected, instead, the relatively higher dose of decitabine had negligible effect on the proportion of Tregs *in vivo* as compared to the vehicle control.

Dysregulation of the innate immune response plays a crucial role in the pathogenesis of MS [29]. In particular, excessive activation of microglia and monocyte-derived macrophages contributes to the development and progression of MS. In the current study, we found that decitabine significantly suppressed the activation of microglia and monocyte-derived macrophages, characterized by lower levels of surface MHC II expression. It is well-established that activated microglia and macrophages produce cytokines and chemokines that recruit additional encephalitogenic T cells to the CNS and exacerbate CNS damage [30–32]. In our study, we found that decitabine significantly inhibited the release of chemokines (CCL2, CCL3, CCL4, CCL5, CXCL10, CCL17, CCL22) from activated microglia and macrophages, which hindered encephalitogenic T cells to the CNS and prevented the disease process of EAE. Increased expression of IL-12, IL-6, IL-23 in activated microglia and macrophages could also direct Th1 and Th17 cell differentiation [5, 33]. Our investigation revealed that decitabine inhibited the upregulation of these cytokines from microglia and macrophages in EAE mice, that may be responsible for inhibiting the differentiation of Th1 and Th17 cells, decreasing the production of IL-17A, IFN-γ from these cells and easing the impairment caused by EAE. Apparently, reduced differentiation of Th1 and Th17 could also be partially attributed to impaired T cell proliferation in the presence of decitabine.

Recent studies demonstrated that mice with decitabine treatment were significantly protected against EAE through inactivation of DNMT1, thus facilitated demethylation of the Foxp3 promoter and induction of Treg differentiation [20, 21]. However, we found that although decitabine treatment significantly increased Foxp3 expression in the *in vitro* differentiation assay, the percentage of Foxp3^+^ Tregs in spleen or thymus showed no obvious changes *in vivo* (Figure 4D, 4E, and Supplementary Figure 5G, 5H). This difference may be explained by the dose and duration of decitabine administration. The regimen we use in this study was 0.25 mg/kg for 2 consecutive weeks, while the previous studies used 0.15 mg/kg for 10 consecutive days or 0.1 mg/kg for 22 consecutive days, respectively [20, 21]. We hypothesized that increased dose of decitabine treatment mainly resulted in the inhibition of T cell proliferation, which led to shrinkage of effector T cell repertoire. Decitabine not only dose-dependently inhibited both CD4^+^ and CD8^+^ T cell proliferation *in vitro*, it decreased the size, weight and total cell numbers of thymus in EAE mice (Supplementary Figure 5A, 5B, 5C). Furthermore, by analyzing the composition of thymocytes in decitabine-treated EAE mice, we found that although decitabine had varying impacts on the percentile of cell subsets (Supplementary Figure 5D, 5E), it nearly decreased the absolute numbers of all cell populations in thymus (Supplementary Figure 5F), including Tregs (Supplementary Figure 5G, 5H). Our results were in consistent with a previous report using decitabine in the treatment of MDS. The major finding was that both high and low dose of decitabine could prevent the progress of MDS through different mechanisms. While low dose decitabine promoted cell differentiation, high dose inhibited tumor cell proliferation through blocking DNA synthesis [22]. However, in the setting of robust immune response such as in our EAE and allo-transplantation models, we have clearly shown in our previous studies that tipping the balance between the Teff versus Treg population was critical to achieve permanent allograft survival [34]. Since Teffs proliferate much faster than Tregs upon TCR ligation or other stimulation, thereby quickly skewing the balance towards the Teff side, it's unlikely that such kind of robust immune response can be controlled by even “super-Tregs”, the number of Tregs may be more critical in such setting. In fact, despite its *in vitro* effect, decitabine merely increased the Treg population *in vivo*. Therefore, we think the such overwhelming effect of decitabine in controlling autogeneic or allogeneic immune response is more than generating “super-Tregs”. So it's rational for us to look at the Teff population which is most critical player in this kind of immune response. Based on the current results, we conclude that inhibition of Teff activation may be the utmost important mechanism to explain the robust therapeutic effect of decitabine in either autogeneic or allogeneic immune reactions.

To further explore the mechanism regarding inhibition of T cell proliferation by decitabine, we found increased transcription of several key cell cycle inhibitors (p15, p16, p21) by promoter demethylation, which inhibited cell cycle progression of CD4^+^ naïve T cells and hindered cell proliferation. More importantly, we unexpectedly found that in addition to its well-known inherent effect of DNMT inhibition, decitabine induced expression of several key cell cycle inhibitors partially via increased expression of a TET family member, TET2, in CD4^+^ T cells upon activation. Further investigation confirmed that the upregulation of TET2 rather than TET1 or TET3 inhibited CD4^+^ T cell proliferation *in vitro*. TET2 has been known to orchestrate the regulation of promoter methylation status by converting 5mc to 5hmc, and function as a tumor suppressor in myeloma and lymphoma [35]. The thylcytosine dioxygenase TET2 could promote DNA demethylation to control the production of IFN-γ and IL-17 in autoimmunity [7], and was required to resolve inflammation by recruiting HDAC2 and repressing transcription of IL-6 through histone deacetylation [36]. A recent study also reported 5-azacytidine could induce the expression of TET2 and reshape morphology of human skin fibroblasts [37]. To our knowledge, this the first time to report that decitabine can control TET expression in CD4^+^ T cells and the importance of TET2 in controlling CD4^+^ T cell proliferation. Collectively, our results suggest decitabine promoted target gene promoter demethylation not only by inhibition of DNMT activity, but via boosting the expression of DNA demethylase TET family member in certain cell types. Interestingly, we identified that knockdown of TET2 could upregulate the expression of p15, p16 and p21, but had minimal effect on that of p27, suggestive of the selectivity of TET2 in controlling gene transcription.

In summary, our study reveals decitabine can be a highly effective approach in the prevention and treatment of EAE, as well as transplant rejection. It also provides a novel mechanism of decitabine on immune regulation which may help to develop potentially innovative treatment strategy for autoimmune diseases and organ transplantation.

## MATERIALS AND METHODS

### Animals

Male C57BL/6 and Balb/c mice aged 6–8 weeks were purchased from the Shanghai SLAC Laboratory Animal Co., Ltd (Shanghai, China). The mice were housed in specific pathogen-free conditions and humanely cared according to the criteria outlined in the National Guideline for the Care and Use of Laboratory Animals. All animal protocols were approved by the Institutional Animal Care and Use Committee of Zhejiang Provincial People's Hospital.

### EAE induction in C57BL/6 mice and decitabine treatment

EAE was induced by subcutaneous injection of 300 μg myelin oligodendrocyte glycoprotein (MOG 35–55) peptide (Sigma, St Louis, MO, USA) emulsified in complete Freud's adjuvant containing 5 mg/ml heat-killed mycobacterium tuberculosis H37Ra (Sigma, St Louis, MO, USA). On the day of immunization and 48 h later, mice were administered intravenously with 200 ng pertussis toxin (List Biological Laboratories, Campbell, CA, USA) in 200 μl of PBS.

Mice received daily intraperitoneal administration of either decitabine (Selleck, USA) (0.25 mg/kg/day) or the same volume of vehicle control for a total of two weeks, starting from day 3 (prevention protocol) or 10 (treatment protocol) after immunization. Mice were observed daily and assessed disease severity according to the following standard criteria [7]: 0 = no obvious clinical sign, 1 = limp tail, 2 = hind limb weakness, 3= complete paralysis of two hind limbs, 4= paraplegia with fore limb weakness or paralysis, 5= moribund or dead.

### Isolation of CNS infiltrating mononuclear cells from EAE mice

To isolate CNS infiltrating cells from EAE mice treated with vehicle or decitabine at day18 after immunization, mice received cardiac perfusion with 50 ml PBS to remove cells from blood vessels under deep anesthesia with 1% pentobarbital sodium. The brain and spinal cord were removed and cut into small pieces in a 70 μm cell strainer placed on a 50 ml tube. The single cell suspension was centrifuged at 390 g for 10 min, and then the cell pellet was resuspended in 10 ml of 37% Percoll and overlaid on 5 ml of 70% Percoll. The sample was centrifuged at 390 g for 20 min at room temperature. After centrifugation, the interphase containing mononuclear cells was carefully collected and washed with PBS for further analysis.

### Cardiac transplantation and decitabine treatment

Cardiac transplantation was performed using microsurgical techniques. Briefly, the donor(Balb/c) ascending aorta was sutured to the recipient(C57BL/6) abdominal aorta in an end-to-side fashion using 10-0 continuous sutures (USSC), and the pulmonary artery to the recipient inferior vena cava (IVC) in the same manner. Prevention of cardiac allograft rejection was induced by intraperitoneal administration of decitabine (0.25 or 0.50 mg/kg/day) or vehicle control for 14 days from day 1 after transplantation. Allograft survival time was determined by daily trans-abdominal palpation of heart beating and confirmed by histological examination.

### Histology

Lumbar spinal cords from decitabine- and vehicle- treated EAE mice at day 18 for prevention protocol and day 30 for treatment protocol after immunization were embedded in paraffin after being fixed in 4% paraformaldehyde (Sigma, St Louis, MO, USA). Samples were then cut into 10 μm slices and stained with conventional hematoxylin and eosin (H&E) to assess infiltration of inflammatory cell or stained with Luxol fast blue (Sigma-Aldrich, St Louis, MO, USA) to evaluate demyelination.

### Enzyme-linked immunosorbent spot (ELISPOT) assay

ELISPOT (e-Bioscience, San Diego, CA) assay was performed according to manufacturer's protocol to assess donor-specific T cell immunity in cardiac transplantation recipient mice by analyzing the production of IFN-γ after rechallenging with allogeneic or syngeneic antigens. Briefly, 96-well plates were coated with capture anti-IFN-γ antibody overnight, 1×10^5^ recipient splenocytes were added to the wells in 200 ml completed RPMI medium. They were stimulated with either irradiated donor bone marrow–derived dendritic cells, or recipient bone marrow–derived dendritic cells as syngeneic controls. No stimulator was added in the negative controls [38]. After incubation (37°C, 5% CO2) for 24 h, biotinylated detection antibody, avidin-horseradish peroxidase and substrate solution were added sequentially followed by incubation and washing after each step. Finally, the spots development in each well was read by an automated Immunospot Analyzer (Cellular Technology, Cleveland, OH).

### Knockdown of TET1, TET2 and TET3 in naïve T cells via shRNA

ShRNAs for knockdown of TET1, TET2, TET3 and GFP-control were constructed into pLvx-shRNA2 (Clontech, Takara) digested with BamHI and EcoRI, and tranfected to HEK293T cells with packaging plasmids (pspax2, pMD2G) using Lipofectamine® 3000 (Thermo, USA). Supernatant was harvested at 24 h and 72 h after transfection and infected anti-CD3ε/CD28 mAb-stimulated naïve T cells. Transfected T cells were then treated with decitabine (10 μM) or vehicle for 96 h. Cells were harvested for real-time PCR to analyze the efficiency of knockdown. ShRNA sequences were detailed in Supplementary Table 2.

### Proliferation and differentiation of naive T cells *in vitro*

Naive T cells were isolated using CD4^+^CD62L^high^ T Cell Isolation Kit (Miltenyi Biotech) from spleen and lymph nodes. Purified CD4+CD62L^high^ T cells (1.5×10^5^ cells/well) were cultured in the presence of anti-CD3ε mAb (2 μg/ml) with or without anti-CD28 mAb (2μg/ml) in complete RPMI medium. Different concentrations of decitabine (1 μM, 5 μM, 10 μM) or vehicle were added at the beginning of T cell proliferation. For differentiation of naïve T cells, IL-12 (10 ng/ml), IL-2 (10 ng/ml), anti-IL-4 (10 μg/ml) and either decitabine or vehicle were added to the culture medium in Th1-skewing conditions. In Th17-skewing conditions, IL-6 (50 ng/ml), IL-23 (20 ng/ml), TGF-β (3 ng/ml), anti-IL-4 (10 μg/ml), anti-IFN-γ (10 μg/ml), and either decitabine or vehicle were added to the culture medium. 4 days later, cells were harvested for FACS, real-time PCR, CFSE or WST-8 (Biotool, USA) assays to evaluate the effect of decitabine on the proliferation and differentiation of naive T cells.

### Flow cytometry

Cell surface staining (CD4, CD8, MHC-II, CD44, CD69, CD62L) (eBioscience) was performed at 4°C for 30 min in the dark. For intracellular cytokine staining (IFN-γ and IL-17A) or intranuclear staining for Foxp3 we first labeled cells with surface markers, fixed, permeabilized, and then stained them with IFN-γ, IL-17A or Foxp3 monoclonal antibody (eBioscience). Stained cells were analyzed by FACS Canto II flow cytometer with Diva software (BD Biosciences), data were processed using FlowJo software (Treestar, Ashland, OR).

### Chromatin immunoprecipitation (ChIP) assays and quantitative real-time PCR

ChIP assays were performed using TET2 (Diagenode), IgG antibody as control (Cell signaling technology), and iDeal ChIP-seq Kit for Transcription Factors (Diagenode) with primer pairs specific for the promoters of *p15*, *p16*, *p21 and p27* genes. Primer sequences are available upon request. For quantitative real-time PCR, total RNA was extracted from CD4^+^ naïve T cells using TRIzol (Invitrogen, Carlsbad, CA, USA) according to the manufacturer's instructions, and reverse transcribed to cDNA with a PrimeScriptTM RT reagent kit (Takara). Quantitative PCR was performed using SYBR Green master mix (Takara) and Applied Biosystems ViiA™ 7 Real-time PCR system. Primer pairs were detailed in Supplementary Table 3.

### Statistical analysis

Data from different groups were compared using the student's t-test, except for the clinical scores of EAE mice, which was performed with the Mann-Whitney U test. P value < 0.05 was considered statistically significant.

## SUPPLEMENTARY MATERIALS FIGURES AND TABLES





## References

[1] Yin QQ, Liu CX, Wu YL, Wu SF, Wang Y, Zhang X, Hu XJ, Pu JX, Lu Y, Zhou HC, Wang HL, Nie H, Sun HD (2013). Preventive and therapeutic effects of adenanthin on experimental autoimmune encephalomyelitis by inhibiting NF-kappaB signaling. J Immunol.

[2] Friese MA, Fugger L (2007). T cells and microglia as drivers of multiple sclerosis pathology. Brain.

[3] Brinkmann V, Billich A, Baumruker T, Heining P, Schmouder R, Francis G, Aradhye S, Burtin P (2010). Fingolimod (FTY720): discovery and development of an oral drug to treat multiple sclerosis. Nat Rev Drug Discov.

[4] Constantinescu CS, Farooqi N, O’Brien K, Gran B (2011). Experimental autoimmune encephalomyelitis (EAE) as a model for multiple sclerosis (MS). Br J Pharmacol.

[5] Saijo K, Glass CK (2011). Microglial cell origin and phenotypes in health and disease. Nat Rev Immunol.

[6] Gao Z, Tsirka SE (2011). Animal models of MS reveal multiple roles of microglia in disease pathogenesis. Neurol Res. Int.

[7] Perry VH, Nicoll JA, Holmes C (2010). Microglia in neurodegenerative disease. Nat Rev Neurol.

[8] Ponomarev ED, Shriver LP, Maresz K, Dittel BN (2005). Microglial cell activation and proliferation precedes the onset of CNS autoimmunity. J Neurosci Res.

[9] Sosa RA, Murphey C, Ji N, Cardona AE, Forsthuber TG (2013). The kinetics of myelin antigen uptake by myeloid cells in the central nervous system during experimental autoimmune encephalomyelitis. J Immunol.

[10] Brosnan CF, Bornstein MB, Bloom BR (1981). The effects of macrophage depletion on the clinical and pathologic expression of experimental allergic encephalomyelitis. J Immunol.

[11] Huitinga I, van Rooijen N, de Groot CJ, Uitdehaag BM, Dijkstra CD (1990). Suppression of experimental allergic encephalomyelitis in Lewis rats after elimination of macrophages. J Exp Med.

[12] Yamasaki R, Lu H, Butovsky O, Ohno N, Rietsch AM, Cialic R, Wu PM, Doykan CE, Lin J, Cotleur AC, Kidd G, Zorlu MM, Sun N (2014). Differential roles of microglia and monocytes in the inflamed central nervous system. J Exp Med.

[13] Ding Z, Mathur V, Ho PP, James ML, Lucin KM, Hoehne A, Alabsi H, Gambhir SS, Steinman L, Luo J, Wyss-Coray T (2014). Antiviral drug ganciclovir is a potent inhibitor of microglial proliferation and neuroinflammation. J Exp Med.

[14] Zhu J, Yamane H, Paul WE (2010). Differentiation of effector CD4 T cell populations (*). Annu Rev Immunol.

[15] Plimack ER, Kantarjian HM, Issa JP (2007). Decitabine and its role in the treatment of hematopoietic malignancies. Leuk Lymphoma.

[16] Floess S, Freyer J, Siewert C, Baron U, Olek S, Polansky J, Schlawe K, Chang HD, Bopp T, Schmitt E, Klein-Hessling S, Serfling E, Hamann A (2007). Epigenetic control of the foxp3 locus in regulatory T cells. PLoS Biol.

[17] Lal G, Bromberg JS (2009). Epigenetic mechanisms of regulation of Foxp3 expression. Blood.

[18] Zheng Q, Xu Y, Liu Y, Zhang B, Li X, Guo F, Zhao Y (2009). Induction of Foxp3 demethylation increases regulatory CD4+CD25+ T cells and prevents the occurrence of diabetes in mice. J Mol Med (Berl).

[19] Guo H, Wang W, Zhao N, He X, Zhu L, Jiang X (2013). Inhibiting cardiac allograft rejection with interleukin-35 therapy combined with decitabine treatment in mice. Transpl Immunol.

[20] Chan MW, Chang CB, Tung CH, Sun J, Suen JL, Wu SF (2014). Low-dose 5-aza-2′-deoxycytidine pretreatment inhibits experimental autoimmune encephalomyelitis by induction of regulatory T cells. Mol Med.

[21] Mangano K, Fagone P, Bendtzen K, Meroni PL, Quattrocchi C, Mammana S, Di Rosa M, Malaguarnera L, Coco M, Magro G, Di Marco R, Nicoletti F (2014). Hypomethylating agent 5-aza-2′-deoxycytidine (DAC) ameliorates multiple sclerosis in mouse models. J Cell Physiol.

[22] Saba HI (2007). Decitabine in the treatment of myelodysplastic syndromes. Ther Clin Risk Manag.

[23] Qin X, Guo BT, Wan B, Fang L, Lu L, Wu L, Zang YQ, Zhang JZ (2010). Regulation of Th1 and Th17 cell differentiation and amelioration of experimental autoimmune encephalomyelitis by natural product compound berberine. J Immunol.

[24] Solvason N, Wu WW, Kabra N, Wu X, Lees E, Howard MC (1996). Induction of cell cycle regulatory proteins in anti-immunoglobulin-stimulated mature B lymphocytes. J Exp Med.

[25] Khanna AK (2005). Reciprocal role of cyclins and cyclin kinase inhibitor p21WAF1/CIP1 on lymphocyte proliferation, allo-immune activation and inflammation. BMC Immunol.

[26] Chow YH, Zhu XD, Liu L, Schwartz BR, Huang XZ, Harlan JM, Schnapp LM (2010). Role of Cdk4 in lymphocyte function and allergen response. Cell Cycle.

[27] Pastor WA, Aravind L, Rao A (2013). TETonic shift: biological roles of TET proteins in DNA demethylation and transcription. Nat Rev Mol Cell Biol.

[28] Scourzic L, Mouly E, Bernard OA (2015). TET proteins and the control of cytosine demethylation in cancer. Genome Med.

[29] Almolda B, Gonzalez B, Castellano B (2011). Antigen presentation in EAE: role of microglia, macrophages and dendritic cells. Front Biosci (Landmark Ed).

[30] Aloisi F (2001). Immune function of microglia. Glia.

[31] Columba-Cabezas S, Serafini B, Ambrosini E, Sanchez M, Penna G, Adorini L, Aloisi F (2002). Induction of macrophage-derived chemokine/CCL22 expression in experimental autoimmune encephalomyelitis and cultured microglia: implications for disease regulation. J Neuroimmunol.

[32] David S, Kroner A (2011). Repertoire of microglial and macrophage responses after spinal cord injury. Nat Rev Neurosci.

[33] Chastain EM, Duncan DS, Rodgers JM, Miller SD (2011). The role of antigen presenting cells in multiple sclerosis. Biochim Biophys Acta.

[34] Tao R, Hancock WW (2008). Resistance of Foxp3+ regulatory T cells to Nur77-induced apoptosis promotes allograft survival. PLoS One.

[35] Solary E, Bernard OA, Tefferi A, Fuks F, Vainchenker W (2014). The Ten-Eleven Translocation-2 (TET2) gene in hematopoiesis and hematopoietic diseases. Leukemia.

[36] Zhang Q, Zhao K, Shen Q, Han Y, Gu Y, Li X, Zhao D, Liu Y, Wang C, Zhang X, Su X, Liu J, Ge W (2015). Tet2 is required to resolve inflammation by recruiting Hdac2 to specifically repress IL-6. Nature.

[37] Manzoni EF, Pennarossa G, deEguileor M, Tettamanti G, Gandolfi F, Brevini TA (2016). 5-azacytidine affects TET2 and histone transcription and reshapes morphology of human skin fibroblasts. Sci Rep.

[38] Tao R, de Zoeten EF, Ozkaynak E, Chen C, Wang L, Porrett PM, Li B, Turka LA, Olson EN, Greene MI, Wells AD, Hancock WW (2007). Deacetylase inhibition promotes the generation and function of regulatory T cells. Nat Med.

