# Supplementary material for: Decitabine inhibits T cell proliferation via a novel TET2-dependent mechanism and exerts potent protective effect in mouse auto- and allo-immunity models
Source: Oncotarget. 2017 May 22;8(34):56802–15. doi: 10.18632/oncotarget.18063 (PMC5593603; doi:10.18632/oncotarget.18063)
Supplement: Supplementary file 1 [file oncotarget-08-56802-s001.pdf]

Decitabine inhibits T cell proliferation via a novel TET2-dependent mechanism and exerts potent protective effect in mouse auto- and allo-immunity models

SUPPLEMENTARY MATERIALS

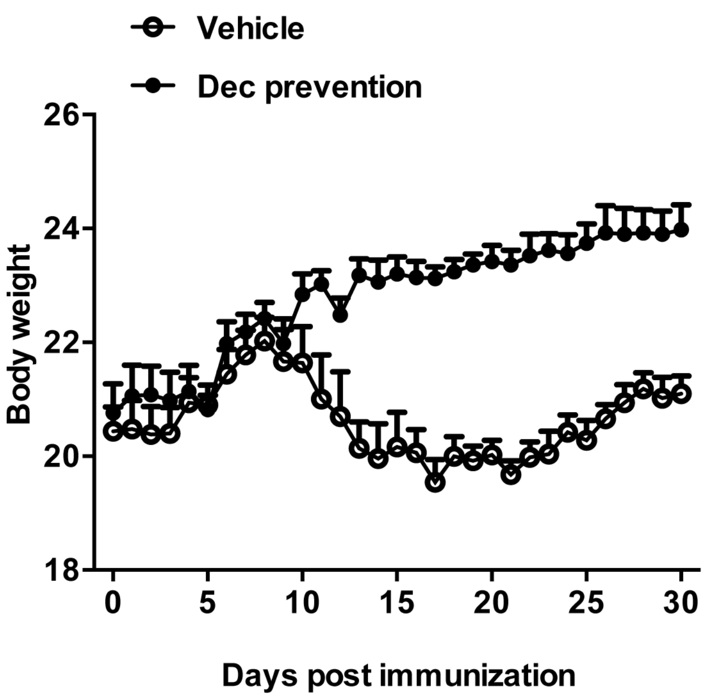

Supplementary Figure 1: Decitabine maintains the weight of EAE mice using the preventive protocol. Body weight gain was observed in EAE mice treated with decitabine or vehicle using the preventive protocol.

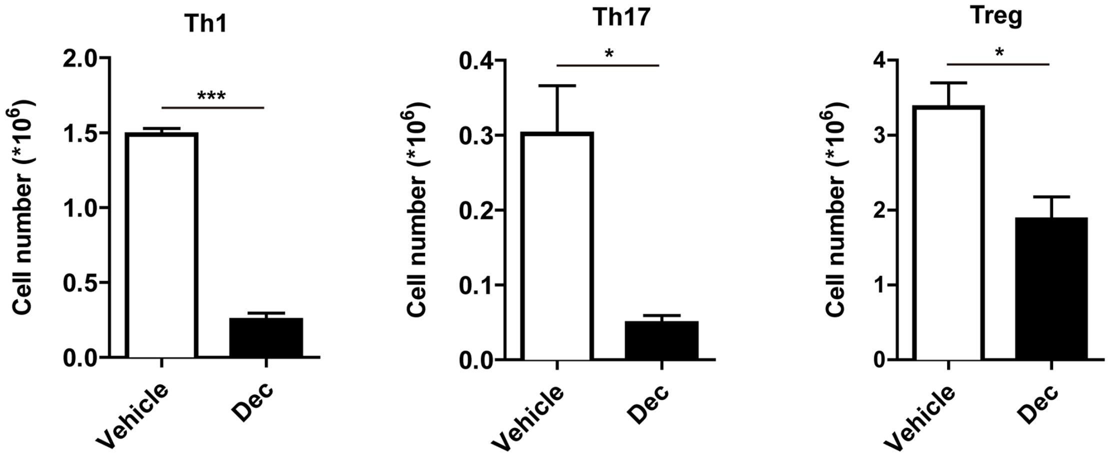

Supplementary Figure 2: Decitabine alters the number of Th1, Th17 and Treg cells in splenocytes from EAE mice. Absolute numbers of Th1, Th17 and Treg cells in splenocytes were summarized. Data were expressed as mean±S.E.M. *$P < 0.05$, ***$P < 0.001$.

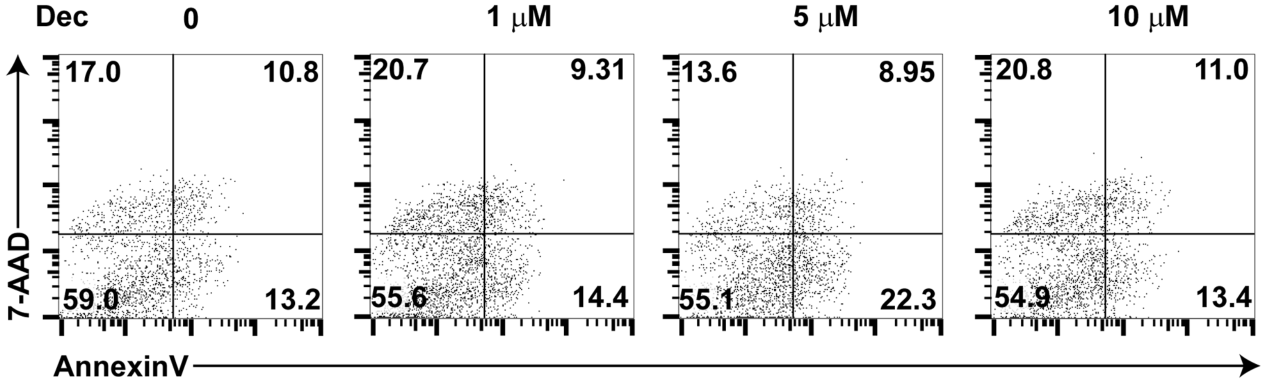

Supplementary Figure 3: The effect of decitabine on the apoptosis of T cells. The effect of decitabine on the apoptosis of T cells stimulated with anti-CD3 plus anti-CD28 mAbs at different concentrations was examined by apoptosis assay kit.

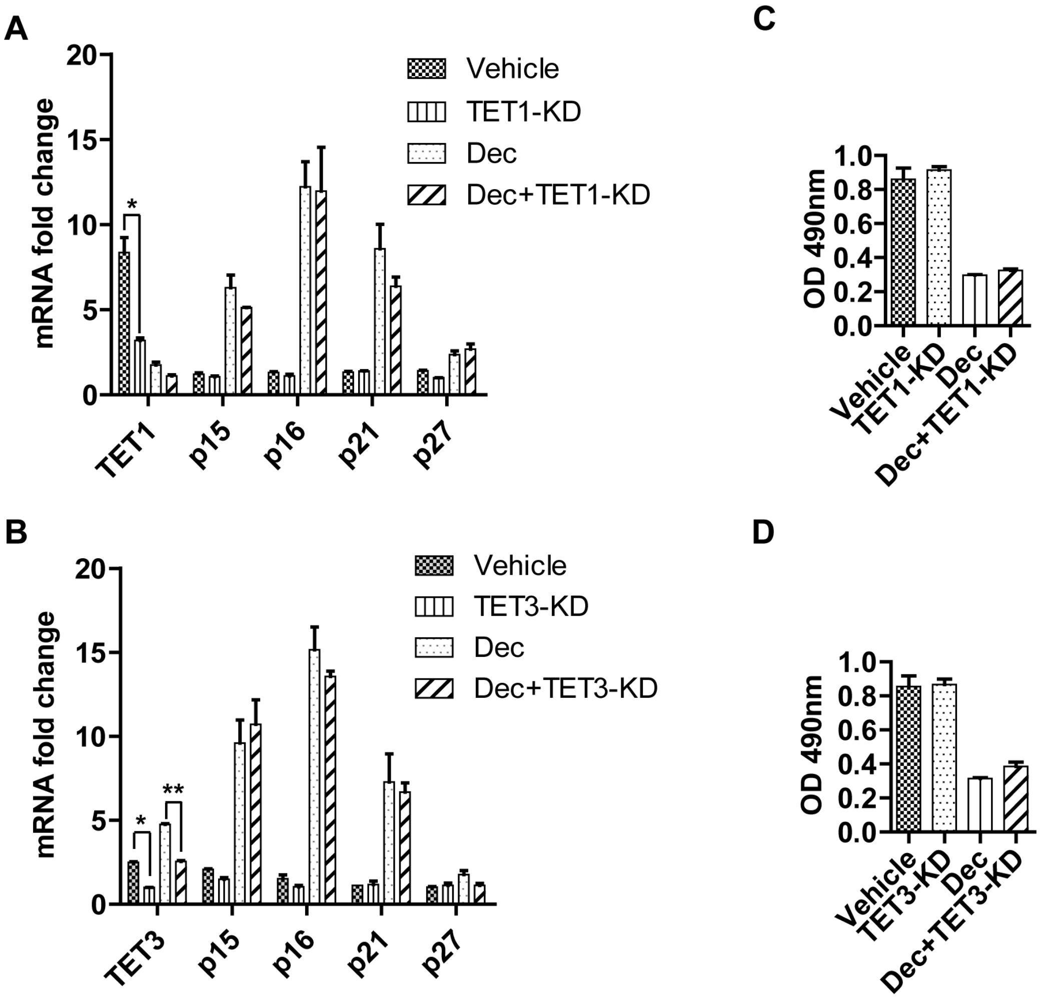

Supplementary Figure 4: The effect of TET1 and TET3 on the proliferation of naïve T cells *in vitro*. Knocking down the expression of TET1 or TET3 in naïve CD4$^+$ T cells, these cells were treated with vehicle or decitabine (10 μM). The different impacts on the transcriptional level of cell cycle inhibitors (p15, p16, p21, p27) **(A, B)** and the rate of cell proliferation **(C, D)** were shown. Data (**A, B, C, D**) were expressed as mean±S.E.M. $*P < 0.05$, $**P < 0.01$.

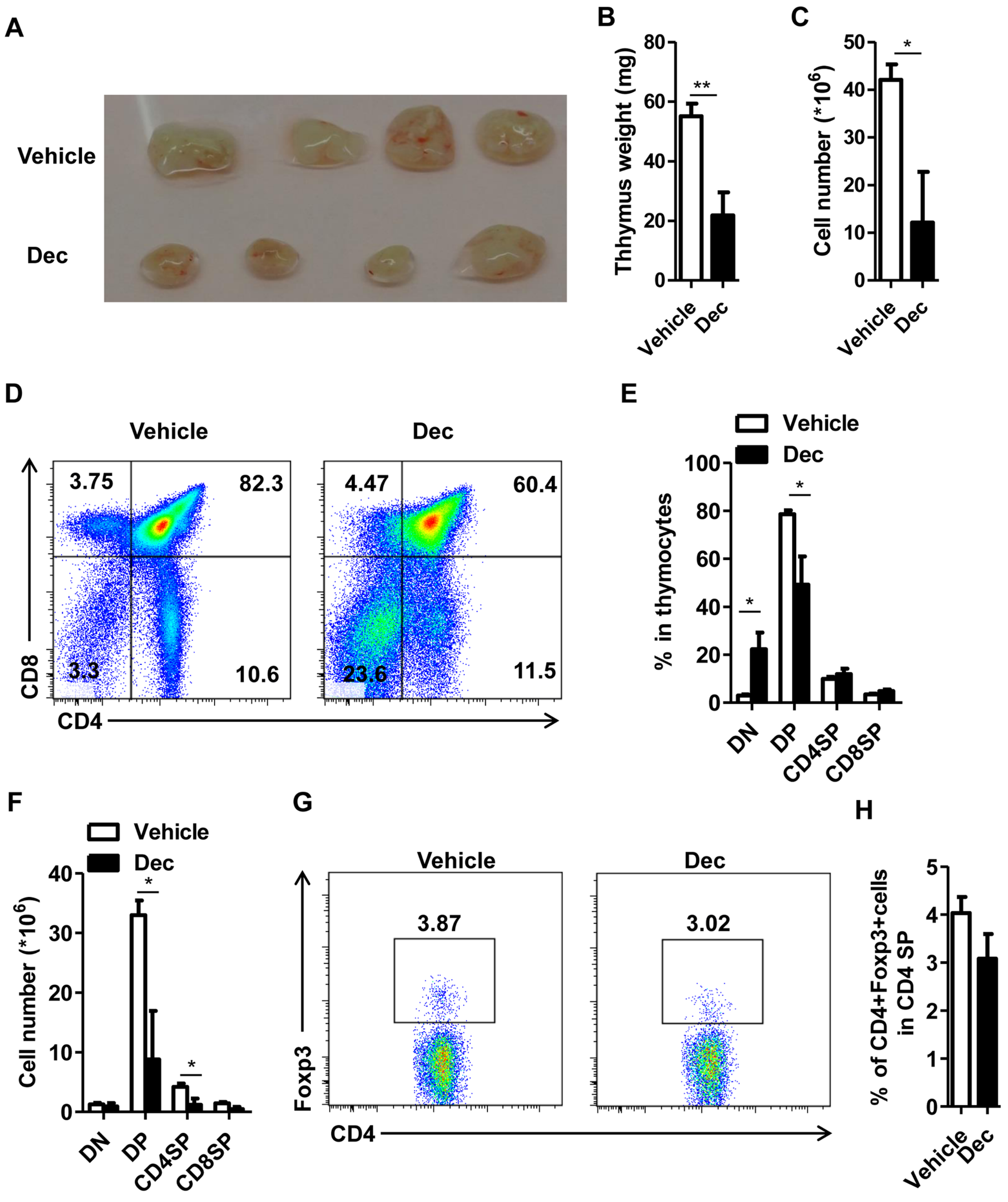

Supplementary Figure 5: Decitabine alters the frequency and number of thymocytes in EAE mice. Thymus was harvested from EAE mice treated with vehicle or decitabine (n=4 mice per group) at day 18 after immunization. The size of thymus was visibly reduced in decitabine-treated EAE mice as compared with vehicle control **(A)**. The weight of thymus and total cell numbers of thymocyte were also measured **(B, C)**. The composition of thymocytes was further analyzed by flow cytometry. Representative plot showed CD4/CD8 subsets of thymocytes in differently treated EAE mice **(D)**. The frequency and absolute numbers of DN, DP, CD4-SP and CD8-SP thymocytes were summarized **(E, F)**. $CD4^{+}Foxp3^{+}$ (nTreg) population in the thymocytes was also analyzed **(G, H)**. Data (**B, C, E, F, H**) were expressed as mean±S.E.M. *$P < 0.05$, **$P < 0.01$.

Supplementary Table 1: Decitabine reduces the absolute numbers of immune cell sub-populations in peripheral blood

	Naïve	Vehicle	Dec
White blood cell $*10^9/L$	2.20 ± 0.10	22.87 ± 5.00	3.05 ± 0.66
Neutrophil $*10^9/L$	0.25 ± 0.15	0.27 ± 0.03	0.35 ± 0.11
Eosinophil$*10^9/L$	0	0	0
Basophil$*10^9/L$	0.05 ± 0.05	0.90 ± 0.47	0.13 ± 0.06
Monocyte$*10^9/L$	0	0.80 ± 0.20	0.45 ± 0.29
Lymphocyte$*10^9/L$	1.85 ± 0.25	20.93 ± 5.17	2.10 ± 0.31
Red blood cell$*10^9/L$	8.64 ± 1.57	8.85 ± 0.17	6.89 ± 1.12
Platelet$*10^9/L$	915.50 ± 260.50	1015.00 ± 57.65	923.00 ± 170.33

White blood cells, particularly the lymphocyte subclasses, were markedly lower in decitabine-treated EAE mice as compared with the vehicle control group.

Supplementary Table 2: ShRNA sequences used for knockdown of TET1, TET2, TET3 and control

Gene	Sequence (5′ ⇒ 3′)
mTet3	GCCTGTTAGGCAGATTGTTCT
mTet2	GGATGTAAGTTTGCCAGAAGC
mTet1	GCTCATGGAGACTAGGTTTGG
Control	GTTCAGATGTGCGGCGAGT

Supplementary Table 3: Primers used in real-time PCR analysis of IL-1β, TNF-α, iNOS, IL-6, IL-12p40, IL-12p35, IL-23p19, CXCL10, CCL2, CCL3, CCL4, CCL5, CCL17, CCL22, p15, p16, p21, p27, CyclinB1, Cdk2, Cdk6, TET1, TET2, TET3 and β-actin.

See Supplementary File 1
