# Supplementary material for: Decitabine inhibits T cell proliferation via a novel TET2-dependent mechanism and exerts potent protective effect in mouse auto- and allo-immunity models
Source: Oncotarget. 2017 May 22;8(34):56802–15. doi: 10.18632/oncotarget.18063 (PMC5593603; doi:10.18632/oncotarget.18063)
Supplement: Supplementary file 2 [file oncotarget-08-56802-s002.doc]

**Supplementary T**able 3. Real-time PCR primers

|  | **Primer** | **Sequence (5'➡3')** |
| --- | --- | --- |
| β-actin | Forward | TGTCCACCTTCCAGCAGATGT |
|  | Reverse | AGCTCAGTAACAGTCCGCCTAG |
| IL-1β | Forward | TTTTTGTTGTTCATCTCGGAGCCTGTAG |
|  | Reverse | GAGCACCTTCTTTTCCTTCATCTTTG |
| TNFα | Forward | CTGTAGCCCACGTCG |
|  | Reverse | TTGAGATCCATGCCGTTG |
| iNOS | Forward | CAGCTGGGCTGTACAAACCTT |
|  | Reverse | CATTGGAAGTGAAGCGTTTCG |
| IL-6 | Forward | TAGTCCTTCCTACCCCAATTTCC |
|  | Reverse | TTGGTCCTTAGCCACTCCTTC |
| IL-10 | Forward | TTGCCTGGTCCTCCTGACTG |
|  | Reverse | GATGTCTGGGTCTTGGTTCT |
| IL-12p40 | Forward | GGAAGCACGGCAGCAGAATA |
|  | Reverse | AACTTGAGGGAGAAGTAGGAATGG |
| IL-12p35 | Forward | CTGTGCCTTGGTAGCATCTATG |
|  | Reverse | GCAGAGTCTCGCCATTATGATTC |
| IL-23p19 | Forward | AGCGGGACATATGAATCTACTAAGAGA |
|  | Reverse | GTCCTAGTAGGGAGGTGTGAAGTTG |
| CXCL10 | Forward | CCAAGTGCTGCCGTCATTTTC |
|  | Reverse | GGCTCGCAGGGATGATTTCAA |
| CCL2 | Forward | AAAAACCTGGATCGGAACCAA |
|  | Reverse | CGGGTCAACTTCACATTCAAAG |
| CCL3 | Forward | CACCCTCTGTCACCTGCTCAA |
|  | Reverse | ATGGCGCTGAGAAGACTTGGT |
| CCL4 | Forward | TTCCTGCTGTTTCTCTTACACCT |
|  | Reverse | CTGTCTGCCTCTTTTGGTCAG |
| CCL5 | Forward | GCTGCTTTGCCTACCTCTCC |
|  | Reverse | TCGAGTGACAAACACGACTGC |
| CCL17 | Forward | TACCATGAGGTCACTTCAGATGC |
|  | Reverse | GCACTCTCGGCCTACATTGG |
| CCL22 | Forward | CTCTGCCATCACGTTTAGTGAA |
|  | Reverse | GACGGTTATCAAAACAACGCC |
| p15 | Forward | CCCTGCCACCCTTACCAGA |
|  | Reverse | GCAGATACCTCGCAATGTCAC |
| p16 | Forward | CGCAGGTTCTTGGTCACTGT |
|  | Reverse | TGTTCACGAAAGCCAGAGCG |
| p21 | Forward | CCTGGTGATGTCCGACCTG |
|  | Reverse | CCATGAGCGCATCGCAATC |
| p27 | Forward | TCAAACGTGAGAGTGTCTAACG |
|  | Reverse | CCGGGCCGAAGAGATTTCTG |
| CyclinB1 | Forward | AAGGTGCCTGTGTGTGAACC |
|  | Reverse | GTCAGCCCCATCATCTGCG |
| Cdk2 | Forward | CCTGCTTATCAATGCAGAGGG |
|  | Reverse | GTGCTGGGTACACACTAGGTG |
| Cdk6 | Forward | GGCGTACCCACAGAAACCATA |
|  | Reverse | AGGTAAGGGCCATCTGAAAACT |
| TET1 | Forward | AGAGAAGACAATCGAGAAGTCGG |
|  | Reverse | CCTTCCGTACTCCCAAACTCAT |
| TET2 | Forward | CTCCCATCAGCCATACAGAACC |
|  | Reverse | CTGACTGTGCGTTTTATCCCT |
| TET3 | Forward | TGCGATTGTGTCGAACAAATAGT |
|  | Reverse | TCCATACCGATCCTCCATGAG |
